# Visible and NIR Light Assistance of the N_2_ Reduction to NH_3_ Catalyzed by Cs-promoted Ru Nanoparticles Supported on Strontium Titanate

**DOI:** 10.1021/acscatal.2c00509

**Published:** 2022-04-12

**Authors:** Yong Peng, Josep Albero, Antonio Franconetti, Patricia Concepción, Hermenegildo García

**Affiliations:** † Instituto Universitario de Tecnología Química CSIC-UPV, Universitat Politècnica de València-Consejo Superior de Investigaciones Científicas, Universitat Politecnica de Valencia, Avda. de los Naranjos s/n, 46022 Valencia, Spain; ‡ Departamento Química Orgánica, Facultad de Química, Universidad de Sevilla, Profesor García Gonzalez 1, 41012 Sevilla, Spain

**Keywords:** photothermal, ammonia synthesis, Cs promoter, titanium perovskite, Ru nanoparticles

## Abstract

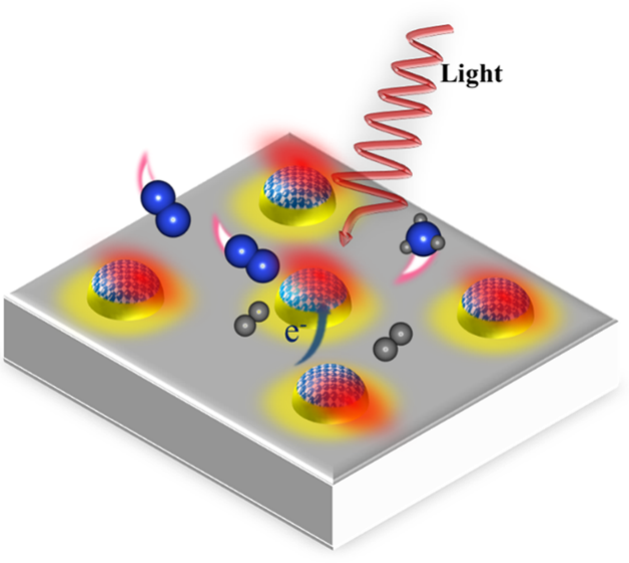

NH_3_ production accounts for more than 1% of the total CO_2_ emissions and is considered one of the most energy-intensive industrial processes currently (*T* > 400 °C and *P* > 80 bars). The development of atmospheric-pressure N_2_ fixation to NH_3_ under mild conditions is attracting much attention, especially using additional renewable energy sources. Herein, efficient photothermal NH_3_ evolution in continuous flow upon visible and NIR light irradiation at near 1 Sun power using Cs-decorated strontium titanate-supported Ru nanoparticles is reported. Notably, for the optimal photocatalytic composition, a constant NH_3_ rate near 3500 μmol_NH_3__ g_catalyst_^–1^ h^–1^ was achieved for 120 h reactions, being among the highest values reported at atmospheric pressure under 1 Sun irradiation.

## 1 Introduction

The most relevant chemical industrial processes (*i.e.*, Bessemer, Gattermann, Haber–Bosch, Fischer–Tropsch, Solvay, *etc.*) are characterized by the enormous energy input required to achieve cost-effective and competitive product formation rates. This is consequence of the unfavorable thermodynamics and sluggish kinetics characteristic of these chemical reactions, making necessary the use of very large pressures and temperatures to achieve adequate production rates. In the context of finding alternative processes to the conventional thermocatalytic reaction, the conversion of inexhaustible and clean sunlight into chemicals and fuels by means of a (photo)catalyst, in the so-called photothermal process, is being increasingly considered an appealing approach to alleviate the high required energy input. The use of solar light should contribute to mitigate the environmental impact caused by the massive consumption of fossil fuels to obtain these enormous amounts of energy.

It is increasingly recognized that the yield of a large variety of chemical reactions of special interest for industry, such as hydrogenations,^1−3^ oxidations,^4,5^ couplings,^6,7^ rearrangements,^8^ among others,^9,10^ can be notably enhanced by the assistance of light. In the conventional photothermal mechanism, light absorption at catalytically active metal nanoparticles (NPs) causes an increase of the local temperature at the nanoscale that is not measurable by conventional macroscopic methods.^11^ This localized temperature increase can result in a much enhanced catalytic activity of the metal NP.^12,13^ This photothermal mechanism causes the selective heating of the absorber, particularly when the thermal conductivity of the support is low, because heat dissipation becomes slower.^14^ When the absorber is a catalytically active site, then, remarkable increases in activity, much higher than those expected according to the macroscopic temperature, can be observed, simply because of the selective conversion of the photon into heat at the proper site. Moreover, in certain cases, it has been proved that chemical reactions can be driven simultaneously by photothermal (selective heating at the NP, not detectable by macroscopic thermocouples) and photocatalytic (photoinduced electron/hole separation) mechanisms.^15,16^

One clear example of photoassisted hydrogenation of an inert molecule is the Sabatier hydrogenation of CO_2_ into CH_4_.^17,18^ The Sabatier reaction is well known to take place at high temperatures above 500 °C in order to achieve production rates convenient for the industry. For this reaction, it has been demonstrated that illumination, even with solar light, increases the CH_4_ formation rate at values required at much higher temperatures and pressures for the pure thermocatalytic process.

Going a step beyond the relatively large number of photothermal and photocatalytic CO_2_ hydrogenation reports,^17−19^ herein, we present an optimized photocatalyst for N_2_ reduction to NH_3_ operating in continuous flow under about 1 Sun illumination. This light-assisted N_2_ fixation has been barely studied so far. Previous studies of photocatalytic N_2_ reduction have focused on semiconductor metal oxides, especially TiO_2_.^20^ However, TiO_2_ presents a 3.2 eV band gap, limiting light harvesting to the UV range, which accounts for less than 5% of the solar light spectrum. Thus, TiO_2_ photocatalysts are not suitable for solar light assistance. Besides metal oxides, Cd-containing dichalcogenides, basically in the form of sulfides, have attracted considerable attention due to their visible light photoresponse. However, the high metal toxicity and poor stability makes Cd and metal chalcogenides far from any practical use. In other study, illumination with up to 47 Suns power, without external heating, of Cs^+^-promoted Ru NPs supported in MgO resulted in a temperature gradient in a thick catalyst bed with a hotter external surface and cooler interior that was found to be a thermodynamic pump favoring NH_3_ formation.^21^

In the present article, we demonstrate that visible and NIR light can assist the N_2_ hydrogenation at atmospheric pressure and temperatures below 400 °C under near 1 Sun (1080 W m^–2^) light intensity using as photocatalyst Cs-promoted ruthenium NPs supported on strontium titanate (Cs_*y*_Ru_*x*_@ST, x referring to Ru loading and y to the Cs/Ru atomic ratio of these elements and ST corresponding to strontium titanate). This photoassisted process will have the advantage of atmospheric pressure and lower macroscopic temperatures than the current industrial process, while using *ca.* 95% of the solar light spectrum. Upon visible and NIR light harvesting, the photocatalyst with optimal composition promotes NH_3_ formation under continuous flow at rates about 68% higher than those obtained in the thermal process under dark conditions and reaching values that are among the highest reported so far for any photocatalytic N_2_ fixation. Moreover, we have studied in detail, using *in situ* spectroscopic techniques, the NH_3_ formation mechanism as well as the role of Cs as the reaction promoter. The Cs-promoted Ru NPs supported on the ST photocatalyst has also demonstrated to be very stable under these reaction conditions for 120 h continuous operation. We believe that this work sets a precedent for the photothermal N_2_ hydrogenation reaction in continuous operation, which is considered a prerequisite for any industrial purpose.

## 2 Results and Discussion

### 2.1 Catalyst Preparation and Characterization

**Scheme 1:**
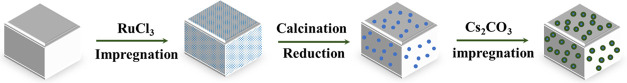
Illustration of the Synthesis of the CsyRu_*x*_@ST Catalyst by the Incipient Wetness Impregnation Method

Ru NPs supported on strontium titanate (Ru_*x*_@ST) containing different Ru loadings (1, 2.5, and 5 wt %) were first prepared to screen out the optimal amount of Ru loading for the N_2_ hydrogenation reaction, and the synthetic procedure is described in detail in the Experimental Section in Supporting Information and illustrated in Scheme 1. Commercial ST particles were impregnated with RuCl_3_ solutions at different concentrations. After drying, the Ru^3+^/ST solids were submitted to calcination at 250 °C and, subsequently, reduced at 350 °C in a H_2_ atmosphere, obtaining the Ru_*x*_@ST catalysts (being X the Ru^3+^ wt %). The amount of Ru loaded in the final Ru_*x*_@ST catalysts was measured by X-ray fluorescence spectroscopy (XRF) and the Ru content of Ru_*x*_@ST catalysts (*x* = 1, 2, and 5) was determined to be 0.94, 2.48, and 4.6 wt %, respectively, based on a previous calibration by XRF using RuO_2_/ST mixtures with different Ru known concentrations.

**Figure 1:**
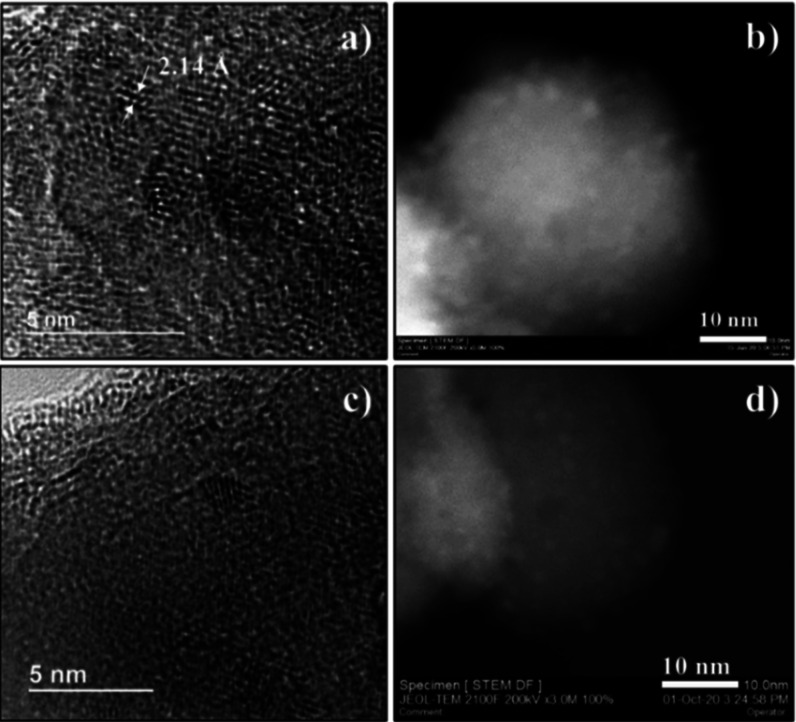
(a) Representative HR-TEM images and (b) ADF-STEM images obtained from the Ru_2_@ST catalyst. (c) Representative HR-TEM images and (d) ADF-STEM images of the Cs_10_Ru_2_@ST catalyst.

The powder X-ray diffraction (PXRD) patterns obtained from Ru_*x*_@ST samples correspond solely to the STO support (see Figure S1 in Supporting Information), and the absence of diffraction peaks from Ru species (neither metallic Ru nor RuO_2_) could be justified by its small particle size and homogeneous distribution on the ST surface. The high dispersity of Ru NPs on the ST surface was further confirmed by high-resolution transmission electron microscopy (HR-TEM) and annular dark-field scanning transmission electron microscopy (ADF-STEM). Figure 1a shows the HR-TEM image obtained from Ru_2_@ST, where highly crystalline NPs, homogeneously deposited on the support surface, can be discerned. A planar distance of 2.14 Å in the crystalline NPs was determined, which matches well with the (002) lattice plane of the metallic Ru phase (PDF #88-1734). ADF-STEM images in Figures 1b and S2 (see Supporting Information) allowed us to measure the dimensions of the Ru NPs, and the average size of Ru_1_@ST, Ru_2_@ST, and Ru_5_@ST was 0.9 ± 0.3, 1.4 ± 0.4, and 1.8 ± 0.5 nm, respectively.

### 2.2 Photothermal Catalytic Ammonia Production Tests

**Figure 2:**
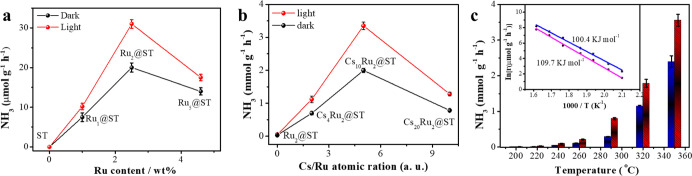
(a) Dependence of the NH_3_ production rate on the amount of Ru loading both under light irradiation (red spheres) and dark conditions (black spheres), (b) NH_3_ production rate obtained from Ru_2.5_@ST with different Cs loadings in both light (red spheres) and dark (black spheres) reaction conditions, (c) NH_3_ production rate obtained from Cs_10_Ru_2_@ST at different reaction temperatures, in both light (red) and dark (blue) conditions. Inset: Arrhenius plot based on the ammonia production rate at different temperatures and the linear fitting in both light (blue) and dark (pink) conditions. The corresponding activation energies are also indicated in the figure. Reaction conditions: 50 mg catalyst, 10 mL min^–1^ N_2_ + 30 mL min^–1^ H_2_, 350 °C, and 0.1 MPa (unless otherwise specified).

Photothermal N_2_ fixation reaction was conducted using the obtained Ru_*x*_@ST samples, targeting to screen out the optimal Ru loading. The reaction was performed using a customized glass flow reactor at 350 °C upon external heating, and light enhancement was evaluated by shifting from dark to light condition (1080 W/m^2^) (see Experimental Section and Scheme S1 for further details of the reaction setup). It is important to note that the thickness of the photocatalytic film (about 1 mm) and the collimated light irradiation should avoid penetration gradients in the catalyst bed (circular 1 cm diameter). In addition, the macroscopic temperature was measured with a thermocouple in contact with the photocatalyst. As shown in Figure 2a, bare ST support exhibited no activity, while 7.02 μmol g^–1^ h^–1^ NH_3_ production was achieved using the Ru_1_@ST catalyst in dark conditions. The production rate further increased to 20.14 μmol g^–1^ h^–1^ with 2.5 wt % Ru loading (Ru_2_@STO) and then decreased to 14.21 μmol g^–1^ h^–1^ when Ru loading reaches 4.6 wt % (Ru_5_@ST). Upon light irradiation, the NH_3_ production rates followed the same trend as that of the dark condition; however, 10.19, 30.90, and 20.35 μmol g^–1^ h^–1^ for Ru_1_@ST, Ru_2_@ST, and Ru_5_@ST, respectively, were obtained. The optimal Ru loading can be explained as a result of two contradictory factors: (a) the increase of Ru NPs as the active sites must promote the N_2_ hydrogenation,^22^ while (b) further increase in the Ru loading results in larger average particle size, and as a consequence, a decrease in active site surface per mass unit. It must be noticed that the light-enhanced activities are over 50%, which, to the best of our knowledge, is the first reported case of photoassisted N_2_ hydrogenation to NH_3_.

To confirm N_2_ as the main source of NH_3_, control experiments using ^15^N_2_-labeled gas were carried out (see Experimental Section in Supporting Information for further details), and the resultant ammonia products were determined by ^1^H NMR. For comparison purposes, a reaction using ^14^N_2_ was also carried out and the obtained products were analyzed by ^1^H NMR. As can be seen from Figure S3, the ^1^H NMR spectrum conclusively confirms N_2_ as the source of NH_3_.^23^

It is worth noticing that the catalytic activity of Ru_2_@ST is below the typical values in the state of the art under similar reaction conditions (see Table S1 in Supporting Information), albeit a 50% of enhancement was achieved. For that reason, further modification of Ru_2_@ST has been carried out. It has been previously reported that the incorporation of alkali or alkaline-earth metal as promoters in some catalysts can remarkably increase the catalytic activity or selectivity in some reactions such as CO_2_ hydrogenation, alkanes dehydrogenation, NO reduction, and N_2_ hydrogenation, among others.^24−27^ Hence, Cs_2_CO_3_ was introduced onto the Ru_2_@ST surface by wet impregnation (see Scheme 1 and Experimental Section in Supporting Information for detailed information). Three Cs_*y*_Ru_2_@ST catalysts with Cs/Ru equal to 2, 5, and 10 were prepared and labeled as Cs_4_Ru_2_@ST, Cs_10_Ru_2_@ST, and Cs_20_Ru_2_@ST, respectively. Figure 1c,d shows the representative HR-TEM and ADF-STEM images of the resultant Cs_10_Ru_2_@ST, which exhibit no particle size or morphology change compared with the Ru_2_@ST sample. PXRD of Cs_*y*_Ru_2_@ST catalysts exhibited only diffraction patterns from ST (Figure S2), which could be explained by the high dispersity of Cs species that can migrate to the vicinity of Ru NPs under a H_2_ atmosphere at the reaction temperature, as reported before.^28^ The migration of Cs species to Ru NPs was lately confirmed by EDX analysis, which revealed the co-presence of Cs and Ru in the high contrast region of the spectrum 1 (Figure S4b in Supporting Information), while no Cs was detected far from the Ru NPs in the low contrast region of spectrum 2. In comparison, no obvious image contrast differences can be observed in samples before the activation (Figure S4a). These differences in contrast are compatible with the coverage of Cs on the whole surface of the support and hence further prove the migration of Cs during the activation treatment. Further evidence of Cs migration to the Ru surroundings has been obtained from EDS analysis of randomly selected regions and elemental mapping of representative STEM images of the Cs@ST sample lacking Ru for which a homogeneous Cs distribution was observed (Figure S4c,d–h, respectively). Preferential Cs deposition near Ru NPs has been previously justified as derived from the higher adsorption energy of Cs for Ru rather than basic support and the same can apply in the present case.^31^

The results of photothermal N_2_ hydrogenation demonstrated a dramatic increase in activity for NH_3_ production in the presence of the Cs promoter, being 3345 μmol g^–1^ h^–1^ under 1080 W/m^2^ light irradiation with the optimal Cs loading (Cs_10_Ru_2_@ST), 100 times higher compared to Ru_2_@ST (Figure 2b). In addition, compared to dark conditions (1989 μmol g^–1^ h^–1^), light irradiation enhanced the NH_3_ production rate by over 68%. To the best of our knowledge, this activity is among the highest values reported so far (see Table S1 in Supporting Information). The dependence of catalytic activity with the Cs loaded follows a similar trend in both light and dark conditions and could be explained also by the simultaneous concurrence of two opposite effects: (1) the presence of Cs species as the promoter can facilitate the nitrogen activation and dissociation (*via infra*), therefore resulting in activity enhancement, while (2) excessive Cs amounts lying next to the Ru NPs can cover partially or completely the Ru NPs, thus decreasing the amount of exposed active sites.^29^ Therefore, the optimal Cs loading must be a compromise between these two factors.

The partial coverage of Cs on the Ru NP surface was investigated by CO chemisorption measurement, and the gas sorption volume versus pressure profiles of Cs_10_Ru_2_@ST and Ru_2_@ST samples are presented in Figure S5. The average crystal size of Ru_2_@ST was 1.464 nm, which is in good agreement with the value obtained from the ADF-STEM image. The calculated Ru surface area was 6.663 m^2^/g, with the metal dispersion of 91.13%. However, the average crystal size obtained in the Cs_10_Ru_2_@ST sample was 3.93 nm, and the surface area has decreased to 2.843 m^2^/g. As confirmed by HR-TEM and AD-STEM images (Figure 1), the Ru particle size remains unchanged after the incorporation of Cs, and thus, the only explanation for the active surface area decreases and the calculated particle size increase is the partial coverage of Cs species on Ru NPs, which is also in agreement with the results obtained from the EDX analysis (Figure S4), while a homogeneous Cs distribution was observed in the absence of Ru after activation (Figure S4c). The partial coverage of Cs on Ru sites can be justified by the fact that the partial reduction of Cs^+^ (*vide infra*) occurred on the Ru sites due to the high concentration of adsorbed H_2_ on its surface.^30−32^ In addition, the Sr component contributes to the high basicity of ST support and thus also favors the migration of Cs species to Ru NPs at high temperature.^33^

### 2.3 Photothermal Effects

To investigate the underlying mechanism of the light enhancement, the reaction was conducted using Cs_10_Ru_2_@ST at temperatures in the range of 200 to 350 °C, either in dark or in light irradiation conditions. As shown in Figure 2c, the NH_3_ production activity exhibited an exponential increase with elevating reaction temperature, both in dark and in light conditions, and over 50% of light enhancement was achieved in all the tested temperature ranges. The activation energies under light and dark conditions were 100.4 and 109.7 kJ/mol, respectively, calculated based on Arrhenius plots (inset in Figure 2c) and equation (eq 1). A similar activation energy value in light and dark conditions indicates the same rate determine step in both cases, suggesting that light-assisted and thermal reactions share the same limiting step in the reaction mechanism.
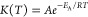
1

**Figure 3:**
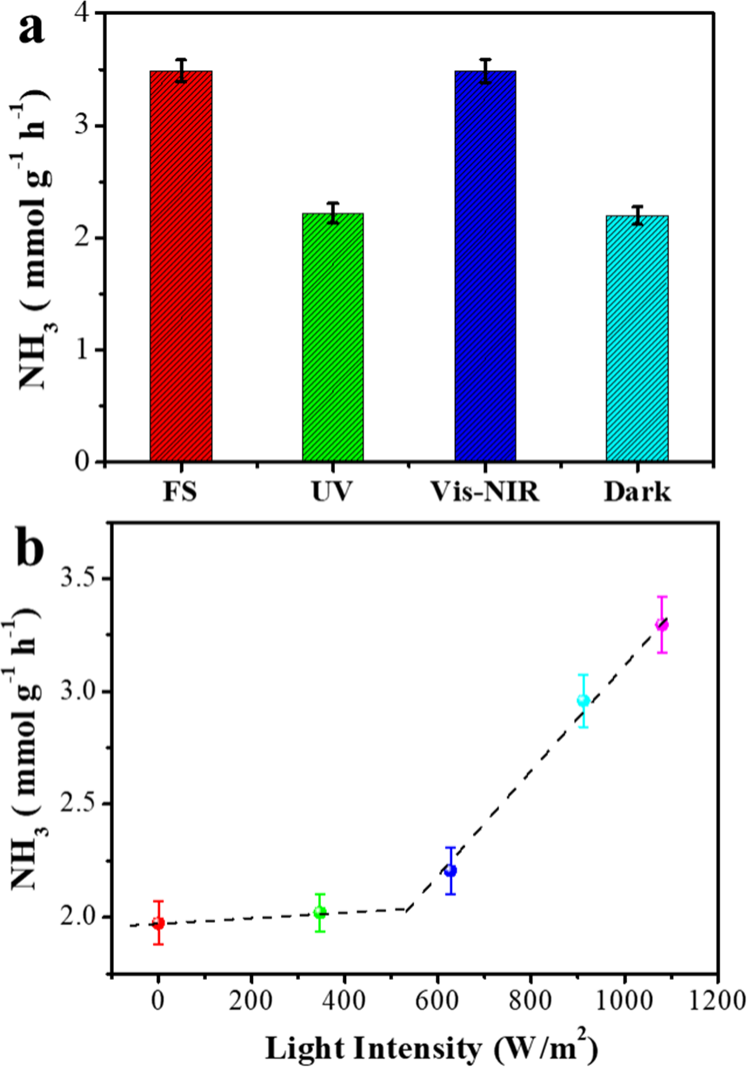
(a) NH_3_ production rate with FS light, UV light, and vis–NIR light irradiation, as well as in dark condition (dark). Light source, 300 W Xenon lamp with FS 1080 W/m^2^. (b) Dependence of the NH_3_ production rate and the irradiation light intensity. Light source, 300 W Xenon lamp with FS 1080 W/m^2^. 50 mg Cs_10_Ru_2_@ST, 10 mL min^–1^ N_2_ + 30 mL min^–1^ H_2_, 350 °C and 0.1 MPa.

The influence of the irradiation wavelength and the light intensity on the NH_3_ production was also investigated. Cs_10_Ru_2_@ST was selectively irradiated with full spectrum (FS), visible, and near infrared (vis–NIR) or ultraviolet (UV) light. As can be observed in Figure 3a, light enhancement originates from the vis–NIR range, while the NH_3_ production rate from UV irradiation can be considered negligible. Furthermore, 515, 610, 695, and 830 nm cut-off filters were applied to evaluate the spectral response. As shown in Figure S6a, the NH_3_ production rates using the 515 and 610 nm filters were almost identical to those of FS irradiation, indicating that light contribution of wavelengths below 610 nm is negligible. On the contrary, 70% of light enhancement has been observed using an 830 nm cut-off filter, suggesting that the light-induced effects mainly originate from the NIR.

Overall, the spectral response of this photothermal NH_3_ system makes it promising for solar light irradiation. The influence of light intensity on the activity was also studied with a 300 W Xe lamp by passing the incident light through neutral density filters before reaching the catalyst. The temperature variations with light intensity in the catalyst surface have been measured (Figure S7), obtaining a maximum temperature increase of 12 °C at the maximum light intensity. Consequently, the external heating temperature controller has been corrected accordingly in order to have constant 350 °C at the different light intensities. Figure 3b shows that the activity increased linearly with the light intensity after reaching 500 W/m^2^, while in the range of 0–500 W/m^2^, negligible activity enhancement was achieved, which indicates that a minimum intensity was required to have a significant activity increase. A similar trend was also observed using the monochromatic 980 nm laser as the light source. In agreement with Figure 2c, this minimum light intensity to form NH_3_ is probably related to the requirement of a minimum local temperature at the Ru NPs to promote N_2_ activation. In addition, as can be seen in Figure S6b, remarkable activity enhancement occurred after the laser power reached 0.5 W, and noticeably, a plateau was reached beyond 1.5 W, which indicates a saturation of the photogenerated carriers. It must be noticed that this conversion is far below the theoretical equilibrium, and even at 450 °C, the decomposition of the ammonia products can be neglected (Figure S8). It is well-known that for pure thermal catalysis, the conversion rates increase with temperature, which can be modulated by the incident light intensity.^34^ In the present case, pure thermal effects induced by simple light-induced heating can be ruled out, as derived from the saturation effect observed in this experiment, and thus, synergistic thermal and photo-induced hot carrier mechanisms are proposed as responsible for the NH_3_ enhancement upon light irradiation, as reported elsewhere.^35^

Diffuse reflectance spectra from Ru_2_@ST and Cs_10_Ru_2_@ST catalysts are presented in Figure S9a in the Supporting Information, showing very similar features. In this, a predominant band in the UV light range (λ < 350 nm), originated from light absorption of ST support, can be observed. Additionally, other absorption bands, starting from 550 nm (inset in Figure S9a), and extended to the NIR range (Figure S9b), can be attributed to light absorption by Ru metal.^18^ Noticeably, the vis–NIR absorption results are coincident with the rate-wavelength dependence observed in Figure S6a, which strongly confirms the light-enhanced NH_3_ production rate originated from the interaction of supported Ru NPs and incident light.

Because 70% of the light enhancement is derived from the NIR range, while the UV light, where the ST has strong response, does not contribute to the NH_3_ production, it is then of interest to study if ST only acts as an inert support for the Ru NPs dispersity. Hence, different supports (TiO_2_ P25, aluminate, and aluminosilicate) loaded with the same amount of Ru and Cs were used as photocatalysts in the NH_3_ production. As observed in Figure S10, the activity of Cs_10_Ru_2_@ST is over two orders of magnitude compared with those using P25, aluminate, and aluminosilicate as the support, despite the lowest surface area it processes (surface area: 46, 71, 275, and 900 m^2^ g^–1^ for ST, P25, aluminate, and aluminosilicate, respectively). In addition, the light enhancement in the case of ST is the highest, being 65, 34, 20, and 31% for ST, P25, aluminate, and aluminosilicate, respectively. Therefore, it is clear that ST is not just an inert support, but can synergistically facilitate the N_2_ reduction reaction. It has been reported that support with higher basicity favors the migration of alkali metals (promoter) to the vicinity of the metal active site during the activation process, and as a consequence, the strongly interaction of the promoter with the active site induces a high catalytic activity.^28^ In the present case, the presence of Sr in the structure enables the ST process to have the highest basicity among those supports; thus it exhibited the highest activity even in dark conditions. Regarding the photothermal enhancement, it has been reported in previous works that supports that possess low thermal conductivity could avoid the heat dissipation from high temperature zones localized in the NPs to the surrounding through the support, and hence a considerable high photothermal effects can be achieved.^14^ This mechanism can explain the high photoenhancement of the NH_3_ rate in the case of ST, as its thermal conductivity is as low as 4.5 W m^–1^ K^–1^ at 350 °C; therefore, ST is an ideal support for the photothermal N_2_ hydrogenation reaction.

### 2.4 Promoter Effects

**Figure 4:**
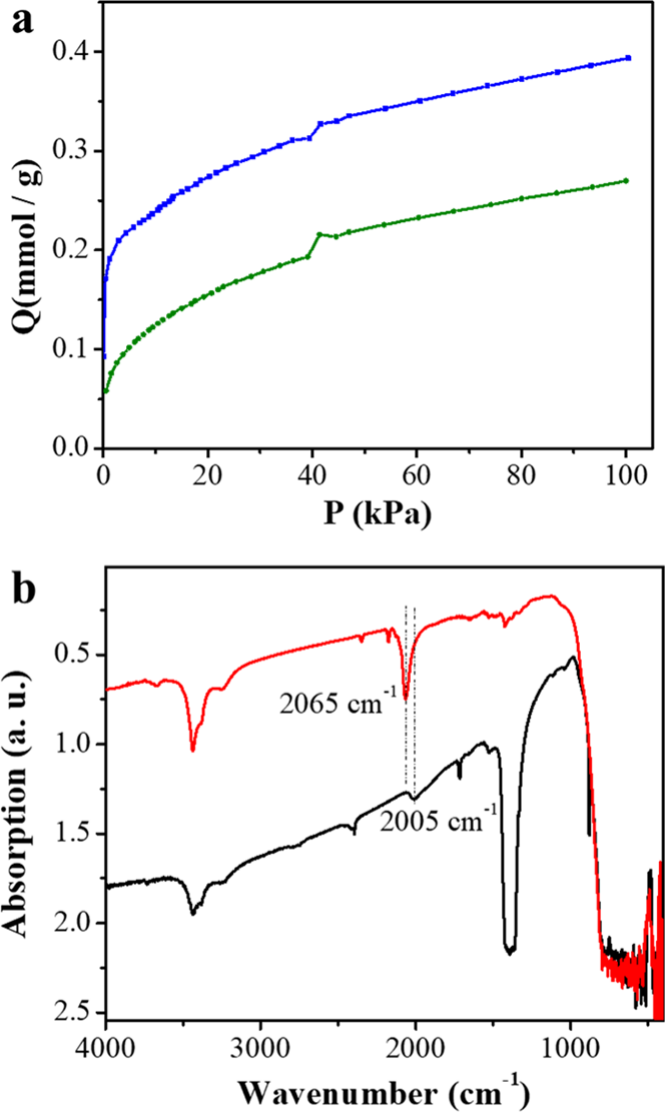
(a) Isothermal CO_2_ adsorption profiles of Cs_10_Ru_2_@ST (blue line) and Ru_2_@ST (green line) obtained at 273 K. (b) FT-IR spectrum of CO chemisorption obtained from Cs_10_Ru_2_@ST (black line) and Ru_2_@ST (red line).

Alkali and alkaline earth transition metals have been used as promoters in reactions, and the promoting effects have been attributed to basicity/acidity changes, structure regulation, and electronic modulation.^29,36−42^ High basicity could promote NH_3_ desorption, as well as electron density migration from the Cs promoter to the Ru active centres. Hence, the basicity in Ru_2_@ST and Cs_10_Ru_2_@ST was evaluated by the measurement of isothermal CO_2_ adsorption. As can be observed in Figure 4a, the CO_2_ adsorption capacity was twofold increased after the incorporation of the Cs promoter. Because no morphology or structure changes have been observed by XRD and ADF-SEM characterization (see Figures 1 and S2), the only explanation can be the increase in the basicity sites. Electron-donating effects from the promoter to the Ru active site were first observed by monitoring CO chemisorption on the catalysts by Fourier transformed infrared spectroscopy (FT-IR). As can be seen in Figure 4b, FT-IR spectra from the Ru_2_@ST catalyst exhibits a peak at 2065 cm^–1^, attributed to C=O vibration from linear adsorbed CO on the Ru surface. This vibration peak shows 55 cm^–1^ shift toward lower wavenumbers in Cs_10_Ru_2_@ST, which is, as reported previously, due to more electron back-donating to the adsorbed CO molecule, and in other words, indicate higher electron density on the Ru NP surface.^43,44^

The electron-donating effect was confirmed by monitoring UV–vis absorption of tetracyanoethylene (TCNE) molecules in the presence of Ru_2_@ST and Cs_10_Ru_2_@ST catalyst. It is well-known that TCNE can accept electrons from the vicinity of electron-rich surfaces, forming e^–^/TCNE complexes that exhibit a characteristic UV–vis absorption peak at ∼300 nm.^45^ As shown in Figure S11a, the intensity of the absorption band centered at ∼300 nm gradually increased with time upon the addition of the Ru_2_@ST catalyst. This result indicates that the TCNE molecule can accept electrons from the adjacent Ru active sites and form e^–^/TCNE complexes. Impressively, the intensity of the peak corresponding to the e^–^/TCNE complex grew 3 times faster in the presence of the Cs_10_Ru_2_@ST catalyst compared to that with Ru_2_@ST, reaching 3 at 11 min (Figure S11b), suggesting the higher electron density on the Cs-decorated catalysts.

X-ray photoemission spectroscopy (XPS) measurements were performed to further confirm the electron density transfer from the Cs species to the Ru active sites. Figure S12 presents the XPS spectrum acquired from Ru_2_@ST and Cs_10_Ru_2_@ST catalysts. As can be seen in Figure S10a, the binding energy of Ru 3d is located in the same range with C 1s and Sr 3p. After deconvolution, clearly distinguished peaks can be seen at 280.53 and 284.73 eV corresponding to metallic Ru. In addition, the deconvolution from Ru 3p + Ti 2p spectra also confirms the reduced Ru species with binding energies of 461.36 and 483.56 eV, together with peaks at 458.46 and 464.16 eV corresponding to Ti^4+^ (Figure S12b). Ru 3d + C 1s + Sr 3p spectra of Cs_10_Ru_2_@ST clearly exhibit the presence of CO_3_^2–^ species; besides, Ru 3d is 0.33 eV shifted to lower binding energy, with components at 280.20 and 284.40 eV (Figure S12c), which can be ascribed to higher electron density of Ru.^28^ This lower binding energy shift was also observed in the Ru 3p spectrum, which shows a binding energy of 461.10 eV for the 3p_3/2_ orbital. Therefore, the XPS results demonstrated a higher electron density of Ru active sites in the presence of Cs as a result of the electron back-donating effects from the Cs promoter. To further demonstrate the underlying reason why the Cs can donate electrons to Ru, XPS analysis of Cs 3d before (Figure S12g) and after (Figure S12e) the H_2_ activation was carried out. As can be observed, the Cs 3d of the sample shows a binding energy of 724.92 eV before activation, while this binding energy is shifted to 725.58 eV after the H_2_ activation. It must be noted that the Cs exhibited inverse chemical shifts to higher binding energy when the oxidation state decreases.^28^ It is well-known that the binding energy of Cs metal is ∼726.1 eV, and hence, it can be concluded that the valence state of Cs species after the activation is between 0 and 1, namely, Cs^δ+^ (0 < δ < 1), which is also in good agreement with the previous report.^28^

Overall, it has been demonstrated that the partially reduced Cs species can have a strong electron-donating ability, transferring electrons to the adjacent Ru sites. Accompanying a partial reduction, Cs_2_CO_3_ species also underwent partial decomposition, as observed from O1s spectra from the Cs_10_Ru_2_@ST sample before and after the activation (Figure S12f,h). As can be seen there, before the activation, the O 1s spectrum is composed of two peaks at 530 and 531.8 eV, which can be attributed to Ti–O and CO_3_^2–^.^46,47^ After the activation, a new component at 532.3 eV (30%) related to −OH species can be observed.^48^ The appearance of this new component after the activation indicates Cs_2_CO_3_ partial decomposition to CsOH.

### 2.5 Reaction Mechanism

N_2_ hydrogenation to NH_3_ involves N_2_ activation and dissociation, as well as the H_2_ splitting to hydride. Therefore, the following control experiments were implemented to make out the key intermediates for the NH_3_ production. Specifically, Cs_10_Ru_2_@ST was Ar-purged in the reaction system for 15 min to remove all the H_2_ gas from the activation process. Then, N_2_ flux was introduced in the reaction chamber at 350 °C for 15 min to form any possible activated N species. Afterward, the reactor was Ar-purged for 15 min to remove the free N_2_ molecules, followed by shifting the gas flow to H_2_. In this way, it is expected that H_2_ will react with the adsorbed N species, forming NH_3_. In the end of this experiment, 7.1 μg of NH_3_ was collected. However, by changing the gas sequence to react the adsorbed hydride with N_2_, only 0.5 μg of NH_3_ was obtained. These results indicate that the key step for the hydrogenation is the N_2_ adsorption and activation, instead of H_2_ adsorption or splitting.

**Figure 5:**
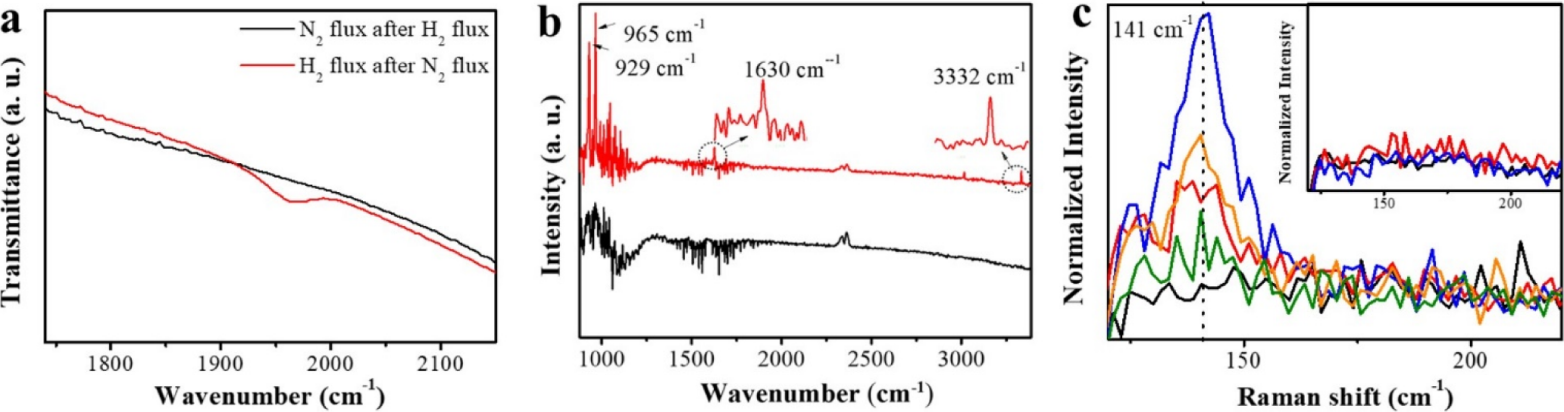
(a) *In situ* FT-IR spectrum of Cs_10_Ru_2_@ST after activation with H_2_ and purging with argon. Subsequently flush with N_2_ followed by H_2_ flux (red line) or H_2_ followed by N_2_ flux (black line). (b) *In situ* FT-IR spectrum in gas after each step in figure (a), that is, the gas phase spectrum of N_2_ after H_2_ flux (red line) and H_2_ after the N_2_ spectrum (black line). (c) *In situ* Raman spectrum of Cs_10_Ru_2_@ST after activation with H_2_ and purging with Ar (black line), followed by N_2_ flux after 10 min (red line) and 20 min (blue line). Afterward, it shifted to H_2_ flow for 10 min (orange line) and 20 min (green line). [Inset: *in situ* Raman spectrum of Ru_2_@ST with the same test steps as that in figure (c); however no signal detected].

Similar results were observed from *in situ* FT-IR characterization. For this study, Cs_10_Ru_2_@ST was first activated at 350 °C for 2 h *in situ* in the chamber, followed by introducing H_2_ and N_2_ (or N_2_ and H_2_) in sequence, and then the FT-IR spectrum was collected. It must be noted that Ar flow was applied for 15 min before introducing N_2_ or H_2_ gas to ensure that the gas phase molecules previously introduced had been totally removed. Figure 5a shows the representative spectrum obtained in this study, where a new peak was observed at 1962 cm^–1^ in the red curve corresponding to the spectrum collected in H_2_ atmosphere after N_2_ flux, whilst no new signal appeared in the reverse gas feeding sequence. The peak at 1962 cm^–1^ cannot be attributed to Ru–H or Ru–H_2_ species as previously reported^43,49,50^ because no signal appeared under the H_2_ atmosphere, even after 8 h holding in such conditions, but appeared only when N_2_ was introduced in the previous step. Therefore, this peak at 1962 cm^–1^ has been assigned instead to adsorbed azide (N=N=N) or oxy-azide (N=N=O) species, as previously reported.^51^ Accordingly, NH_3_ was detected in the FT-IR chamber gas phase only in the H_2_ atmosphere after N_2_ flux, with featured peaks 3332, 1630, 965, and 929 cm^–1^, as marked in Figure 5b. It must be highlighted that this result is coincident with that previously performed in the reactor (*vide ante*). Combining the results in Figure 5a,b, it can be concluded that the produced NH_3_ derives from the nitrogen species detected at 1962 cm^–1^, as shown in Figure 5a, in the *in situ* FITR experiments and is attributed to azide or oxo-azide species.

The formation of adsorbed N_2_ species was further studied by *in situ* Raman spectroscopy using Cs_10_Ru_2_@ST and Ru_2_@ST catalysts, and the results are shown in Figure 5c. As can be observed in Figure 5c, N_2_ flux on Cs_10_Ru_2_@ST after the *in situ* activation results in a new peak at 141 cm^–1^, which has been attributed to δNRuO species formed on the Cs_10_Ru_2_@ST surface,^52^ and the intensity increases with time in contact with N_2_. Then, the intensity gradually decreased with time after shifting the gas to H_2_, which can be ascribed to the consumption of δNRuO species by H_2_, forming NH_3_ products. On the contrary, no signal was observed in the case of Ru_2_@ST (Inset in Figure 5c), indicating that the N_2_ could not be easily bonded to the Ru surface or activated, while the presence of the Cs promoter can increase the interaction ability of Ru with N_2_ and therefore facilitate the N_2_ activation.

The strong interaction of Ru with N_2_ in the case of Cs_10_Ru_2_@ST was also confirmed by thermo-programmed desorption (TPD) experiments. In a first test, the Cs_10_Ru_2_@ST sample was subjected to H_2_ at 350 °C for 2 h and then cooled to room temperature with the purge of Ar before H_2_ TPD measurement. Alternatively, Cs_10_Ru_2_@ST was in contact with a mixture of H_2_ and N_2_ flow (3:1 in volume ratio) at 350 °C for 2 h after the activation, and then H_2_ TPD measurement was performed after the system cooled to room temperature under Ar flow. As can be observed in Figure S13 in Supporting Information, the Cs_10_Ru_2_@ST exhibited an intense H_2_ desorption peak from 400 to 600 °C when the measurement followed after the activation step, which demonstrates that the H_2_ can be adsorbed efficiently on the surface of Ru or even penetrate deep in its subsurface. However, the desorption peak was drastically decreased when treating Cs_10_Ru_2_@ST by the H_2_/N_2_ mixture, indicating that the N_2_ molecule could strongly interact with Ru active sites, which should compete to H_2_ adsorption, and therefore, results in low H_2_ desorption intensity.

**Scheme 2:**
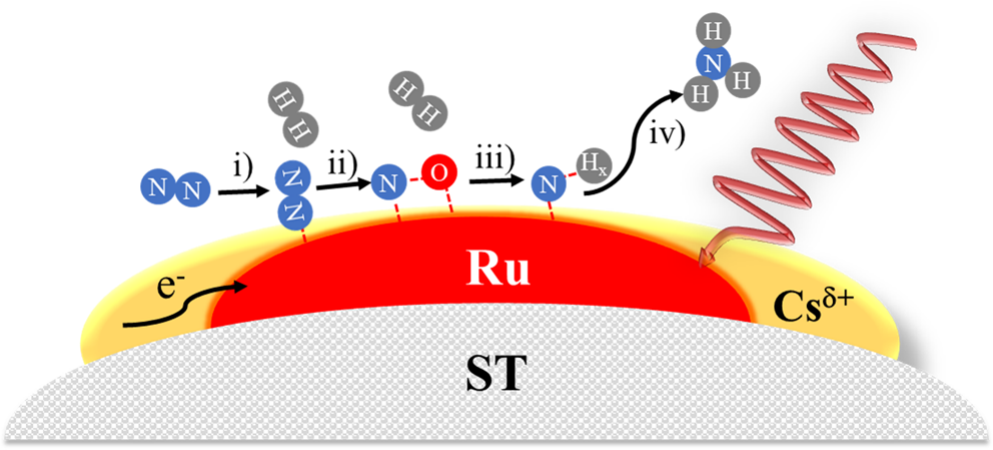
Illustration of the Proposed Mechanism: (i) N_2_ Adsorption and Activation, (ii) N_2_ Dissociation and Formation of Intermediate Species, (iii) Hydrogenation of Activated Nitrogen Species, and (iv) NH_3_ Formation and Desorption

To sum up, results from the abovementioned studies revealed that the partially reduced Cs promoter can be responsible not only for the electron density transfer to Ru sites, but also for the formation of oxy-azide-related species, which can be then sequentially hydrogenated to the final NH_3_ product. Moreover, upon light irradiation, Ru NPs absorb vis-to-NIR light, generating hot electrons and elevating the local temperature at the NP, thus, greatly assisting in the activation of dinitrogen molecules. Subsequently, the activated nitrogen is sequentially hydrogenated and, eventually, ammonia is produced and released. A scheme of the proposed mechanism is presented as Scheme 2.

### 2.6 Catalyst Stability

The stability of the Cs_10_Ru_2_@ST catalyst was evaluated by conducting the N_2_ fixation reaction at 350 °C continuously for 120 h under 300 W Xenon lamp irradiation. The temporal profile of the ammonia production rate is shown in Figure S14. As shown in the graph, a slight decay of the activity occurred during a continuous 120 h reaction, demonstrating the high stability of the Cs_10_Ru_2_@ST catalyst. More specifically, it can be found that only 10% of activity is lost in 60 h compared to the fresh sample, and additional 10% activity loss at 120 h. In addition, it can be observed from HR-TEM images (Figure S15) that the Ru particle size and dispersity of Cs_10_Ru_2_@ST remained unchanged before and after 120 h reaction, further confirming the high stability of the Cs_10_Ru_2_@ST catalyst.

## 3 Conclusions

This study reports the visible and NIR light-driven improvement in the N_2_ hydrogenation reaction to NH_3_, when optimized Cs-decorated strontium titanate-supported Ru NPs is used as a catalyst. Isotopic ^15^N labeling experiments firmly confirm N_2_ as the origin of NH_3_. The NH_3_ production is 68% increased upon 1080 W m^–2^ irradiation (corresponding to about 600 W m^–2^ of effective light power) and depends on the wavelength, the NIR radiations being more efficient than wavelengths in the visible range, while UV light does not contribute to the NH_3_ production rate. This makes the photocatalyst very appropriate for the use of solar light. Mechanistic studies indicate that the reaction occurs upon light absorption by Ru active sites causing a localized temperature increase (photothermal mechanism) and that ST plays a key role due to its basicity and low thermal conductivity. *In situ* FTIR experiments have been carried out to determine the reaction mechanism. Considering that upon illumination the Cs_10_Ru_2_@ST photocatalyst reported here is among the most efficient catalysts reported so far for NH_3_ formation, the present finding opens the door for further exploration of light assistance to ammonia synthesis and helps in the development of more advanced photo-responsive catalysts.

## Supporting Information Available

Catalyst preparation and characterization, reaction setup and test methodology, catalyst performance and stability data, XRD, ^1^H NMR, EDS and mapping, HR-TEM, CO chemisorption, UV–vis absorption, XPS, TPD data, and a table list of the state-of-the-art of the catalysts for nitrogen fixation (PDF)
