# Supplementary material for: Visible and NIR Light Assistance of the N2 Reduction to NH3 Catalyzed by Cs-promoted Ru Nanoparticles Supported on Strontium Titanate
Source: ACS Catal. 2022 Apr 12;12(9):4938–46. doi: 10.1021/acscatal.2c00509 (PMC9087182; doi:10.1021/acscatal.2c00509)
Supplement: Supplementary file 1 — cs2c00509_si_001.pdf [file cs2c00509_si_001.pdf]

Supporting Information

Visible and NIR-light assistance of the N_2 reduction to NH_3 catalyzed by Cs-promoted Ru nanoparticles supported on strontium titanate.

Yong Peng,[a] Josep Albero,[a] Antonio Franconetti,[b] Patricia Concepción [a] and Hermenegildo García*[a]*

[a] Instituto Universitario de Tecnología Química CSIC-UPV, Universitat Politècnica de València-Consejo Superior de Investigaciones Científicas, Universitat Politecnica de Valencia, Avda. de los Naranjos s/n, 46022 Valencia, Spain

[b] Departamento Química Orgánica, Facultad de Química, Universidad de Sevilla, Profesor García Gonzalez 1, 41012 Sevilla, Spain.

* Corresponding Authors at: Instituto Universitario de Tecnología Química CSIC-UPV, Universitat Politècnica de València-Consejo Superior de Investigaciones Científicas, Universitat Politecnica de Valencia, Avda. de los Naranjos s/n, 46022 Valencia, Spain.

E-mail address: joalsan6@upvnet.upv.es; hgarcia@qim.upv.es

Experimental Section

Chemicals

All chemicals were used as received without further purification. Ruthenium chloride hydrate ($RuCl_3 \cdot xH_2O$, 98 %), strontium titanate ($SrTiO_3$), sodium nitroprusside dihydrate ($Na_2[Fe(CN)_5NO] \cdot 2H_2O$, 99 %), phenol (99.9 %), sulfuric acid (95-98 %, ACS REAGENT), cesium carbonate (Cs_2CO_3, 99 %), $^{15}N_2$ (98 % atom purity) and sodium citrate tribasic ($HOC(COONa)(CH_2COONa)_2$, 99 %) were purchased from Sigma Aldrich. Sodium hypochlorite solution (NaClO, 11-14 wt%) was obtained from Fisher Chemical™. Nitrogen gas (N_2, 99.999 %) and hydrogen gas (H_2, 99.999 %) were purchased from Abelló Linde SA. Absolute ethanol (analytical grade) and acetone (analytical grade) were supplied by Scharlau. MilliQ water was prepared using an IQ 7000 purifying system. NaOH micro-pearls (98 %) were purchased from ACROS.

Characterization

Powder X-ray diffraction (PXRD) patterns were recorded with a Shimadzu XRD-7000 diffractometer by using $Cu_{\kappa\alpha}$ radiation (λ=1.5418Å), operation at 40 kV and 40 mA at a scanning speed of 10° per min in the 2–90° 2θ range. The content of ruthenium was determined X-ray flourenscence characterization (XRF, Philips MiniPal 25 fm instrument). Diffuse reflectance UV/Vis spectra (DRS) in the range of 200 - 2000 nm were collected in a Varian Cary 5000 spectrophotometer from Varian. ^{1}H NMR was recorded with a Bruker AV400 (400 MHz) spectrometer. X-ray photoelectron spectra (XPS) were measured on a SPECS spectrometer equipped with a Phoibos 150 MCD-9 detector using a non-monochromatic X-ray source (Al) operating at 200 W and the activation process was conducted in-situ at 350 °C for 2 h before the measurement. Before the spectrum collection, samples were evacuated in the prechamber of the spectrometer at 1×10^{-9} mbar. The measured intensity ratios of the components were obtained from the area of the corresponding peaks after nonlinear Shirley-type background subtraction and corrected by the transition function of the spectrometer. Because of the overlaps of Ru 3d and C 1s, the calibration was done referenced to Sr 3d 5/2 (133.1 eV) and Ti 2p3/2 (458.4 eV). The *in-situ* FTIR spectra were collected with a Bruker "vertex 70" and a Thermo Nicolet 8700 spectrometer equipped with a DTGS detector (4 cm–1resolution, 32 scans). An IR cell allowing in situ treatments under controlled atmospheres and temperatures from 25 ºC to 500 ºC has been connected to a vacuum system with gas dosing facility. Self-supporting pellets (ca. 10 mg cm^{-2}) were prepared from the sample powders and treated in hydrogen flow (30 ml min^{-1}) at 350 ºC for 2 h before the test. *In-situ* Raman spectra were obtained with an "in via" Renishaw spectrometer, equipped with an Olympus microscope. The samples were treated with H_2 at 350 °C for activation *in-situ* before the spectrum collection. The H_2 desorption was monitored with a thermal conductivity detector (TCD) and a mass-spectrometer following the characteristic mass of H_2 at 15 a.m.u. The CO_2 adsorption isotherms in the low-pressure range were measured by using a Micromeritics ASAP 2010 instrument using ~200 mg of catalyst placed in a sample holder, which was then immersed into a liquid circulation thermostatic bath for precise temperature control. Before each measurement, the sample was treated overnight at 350 °C under vacuum and then measured at 0 °C.

Ru NPs dispersity in Cs_yRu_x@ST samples were evaluated by CO monolayer Chemisorbed volume (Vm) using the double isotherm method on a Quanta chrome Autosorb-1C equipment. Before the measurement, the catalysts were activated in situ by 30 mL / min of H_2 flow at 350 ºC for 2 h. Afterwards, the catalysts were degassed under vacuum (1333x10^{-3} Pa) for 2 h at 350 ºC. Then, pure CO was introduced and the first adsorption isotherm (i.e. the total CO uptake) was measured. After evacuation at at 350 ºC, the second isotherm (i.e. the reversible CO uptake) was taken. The volume of chemisorbed CO (V_m) was thus obtained by subtracting the two isotherms. The monolayer uptake (Nm) can be obtained through the following equation:

N_m=44.61 V_m **Equation S1**

Where, N_m is in μmol g^{-1} and V_m is in cc g^{-1}.

The Active Surface Area (ASA) can be obtained through **equation S2**.

ASA=(N_mSA_m)/166 **Equation S2**

Where, S is the number of surface atoms covered by each chemisorbed gas molecule (assuming a stoichiometry of Ru/CO=1 in this study), and A_m is the cross-sectional area occupied by each active surface atoms. ASA is in m^2 per gram of sample.

The percent metal dispersion (D) is calculated from equation S3.

D=(N_mSM)/100L **Equation S3**

Where M and L are the molecular weight and percent loading of the supported metal.

The pressure range studied was 0.5-11 x 104 Pa. The mean Ru^0 diameter (d) was determined from chemisorption data assuming spherical geometry for the metal particle according to the procedure described by Anderson.[26]

Catalyst preparation

Preparation of Ru_x@ST. Strontium titanate (ST) supported ruthenium nanoparticle catalysts were prepared by incipient wetness impregnation method. Typically, 1 g of commercial ST (with average particle size 30 nm) was dispersed in 20 mL milli-Q water by strong sonication for 30 mins, followed by adding the wanted amount of $RuCl_3$. Then, the mixture was subjected to bath sonication for about 30 mins until all ruthenium species were homogeneously adsorbed on the ST surface. Afterwards, water was slowly evaporated from the mixture under gently stirring at 70ºC and the as-obtained solid was

further dried in an oven at 100°C overnight. Then, the sample was subjected to calcination at 250°C and reduction at 350°C for 2 h in sequence, with a heating ramp 5 °C / mins for both processes. The catalyst was ready for test after being thoroughly washed with milli-Q water and then dried at 100°C overnight.

Preparation of Cs_yRu_2@ST. Cesium decorated Ru_2@ST catalysts were synthesized by simply impregnating Ru_2@ST catalyst into Cs_2CO_3 solution. Specifically, 500 mg of Ru_2@ST was dispersed in 5 mL milli-Q water by sonication before a given amount of Cs_2CO_3 (catalysts with atomic ratio of Cs: Ru=2, 5, 10 were prepared in this study, and the corresponding value of y=4,10 and 20, respectively) was added to the dispersion. Then, water was removed from the mixture by slowly evaporating at 70 °C and the samples were further dried in an oven at 100 °C overnight. Samples were activated at 350 °C under H_2 flow for 2 h before characterization unless otherwise specified.

Photothermal N_2 hydrogenation test

Photothermal N_2 fixation reaction to ammonia was performed with a customized fixed-bed flow reaction system (as shown in **Scheme S1** in supplementary information). Specifically, 50 mg catalyst was loaded on the fused quartz frit that fixed inside the borosilicate glass tube reactor with an inner diameter of 10 mm and the thickness of the catalyst is around 1 mm. External heating was applied by a heating ribbon that wrapped on the external surface of the tube reactor and the temperature was controlled by a *K*-type thermocouple. Light irradiation was supplied by using a 300 W xenon lamp (1080 W/m^2) and was introduced to the reactor by inserting the optical fiber into the quartz tube that was mounted on the top of the reactor. The spot of the light source that reach the catalyst surface was 10 mm in dimeter thus ensured the uniformity of the light irradiation on the whole catalyst surface. Before each batch test, the catalyst was activated with H_2 for 2 h at 350 °C and the effluent of the last 30 mins of the activation process was collected and analyzed to rule out any possible NH_3 contamination from the system or the catalyst. The feeding gases (H_2 30mL / min and N_2 10mL /min) were purified by passing through 5 mM H_2SO_4 acid and milli-Q water in sequence and dried with $CaCl_2$ before introducing into the reactor. The produced NH_3 was trapped by 5mM H_2SO_4 acid (reservoir 1 and 2) and then quantified by indophenol blue method.

$^{15}N_2$ isotopic experiment

$^{15}N_2$ isotopic experiment was performed to confirm the N_2 source of the NH_3 product and was conducted with the above-mentioned glass flow set-up by slight modification. Specifically, a rubber suction bulb was used to collect the inlet and the outlet of the flow reaction set-up, forming a closed circulation system. After being thoroughly purged with argon, a stoichiometric amount of H_2 and $^{15}N_2$ was introduced into the system. The mixture gas was manually circulated inside the system for 30 mins under reaction conditions and $^{15}NH_3$ trapped in reservoir 1 was analyzed by ^1H NMR.

Ammonia quantification

NH_3 was quantitively determined by indophenol blue method.[1] To consolidate the reliability of this colorimetric method, ^1H NMR quantification was complemented with DMSO as the internal standard. For NH_3 quantification with indophenol method, to 5 mL reservoir 1 solution were added in sequence 200 μL phenol/ethanol solution (100 mg mL^{-1}), 200 μL sodium nitroprusside aqueous solution (0.5 wt %) and 500 μL oxidating solution (0.25 M NaOH solution containing 20 wt% sodium citrate and 1 wt % sodium hypochlorite). After being kept in dark for 3 h, the light absorbance at 640 nm of the resultant blue solution was measured by UV-vis spectroscopy (The parent solution in reservoir 1 was diluted before the addition of indophenol reagent to ensure the absorption at 640 nm in the calibration range). The relationship of the NH_3 concentration and the resultant indophenol blue absorption at 640 nm was established by using a group of known-concentration NH_4Cl solution (C_{NH3} =0, 0.1, 0.5, and 1 ug/mL) and the calibration curve is shown in **Figure S16** in the supplementary information. For the quantitative NH_3 measurement with ^1H NMR spectroscopy, 500 μl of the solution collected in reservoir 1 was mixed with 100 ul deuterium oxide, together with known concentration DMSO as the internal standard. The amount of NH_3 was then obtained based on the calibration with known-concentration of $(NH_4)_2SO_4$ and DMSO standard solution. A representative ^1H NMR spectrum is presented in **Figure S17**.

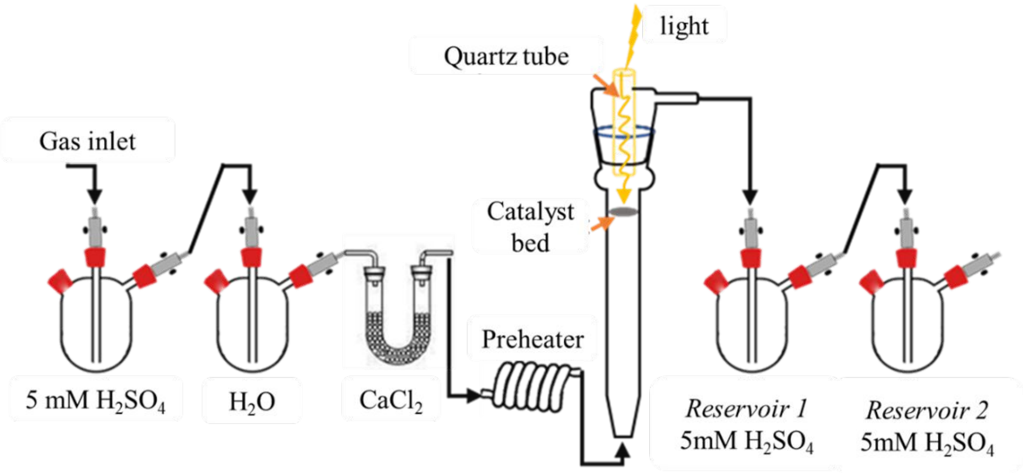

Scheme S1. Illustration of the synthetic photothermal N_2 fixation set up.

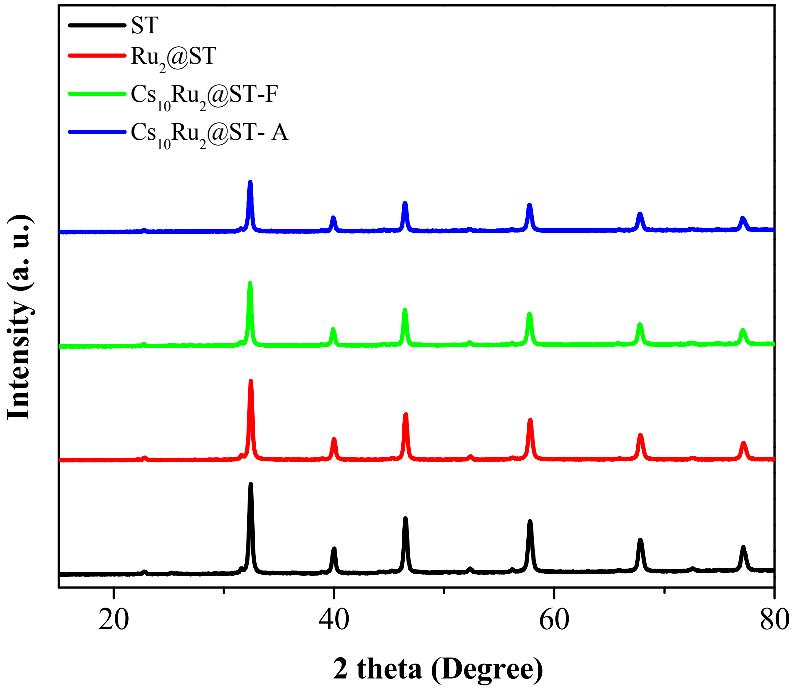

Figure S1 X-ray diffraction patterns obtained from ST (black line), Ru_2@ST (red line), the as-obtained $Cs_{10}Ru_2$@ST (green line), and $Cs_{10}Ru_2$@ST after activation (blue line).

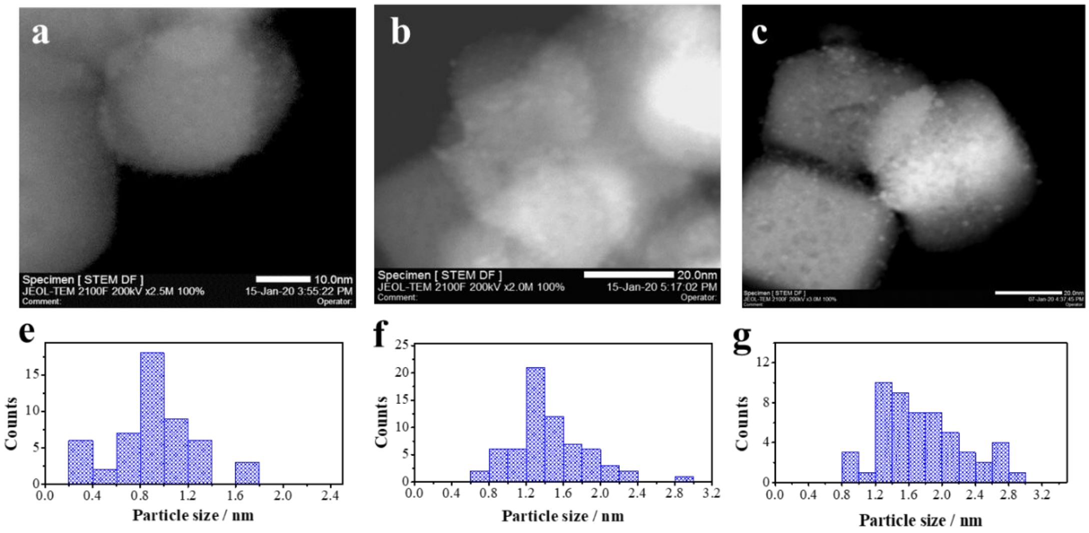

Figure S2 Representative ADF-STEM images obtained from Ru_1@ST **(a)**, Ru_2@ST **(b),** and Ru_5@ST **(c)**. Figure **(e)**, **(f)** and **(g)** show the statistic particle size distribution of 1 %, 2.5 % and 4.6 % Ru loaded sample, respectively.

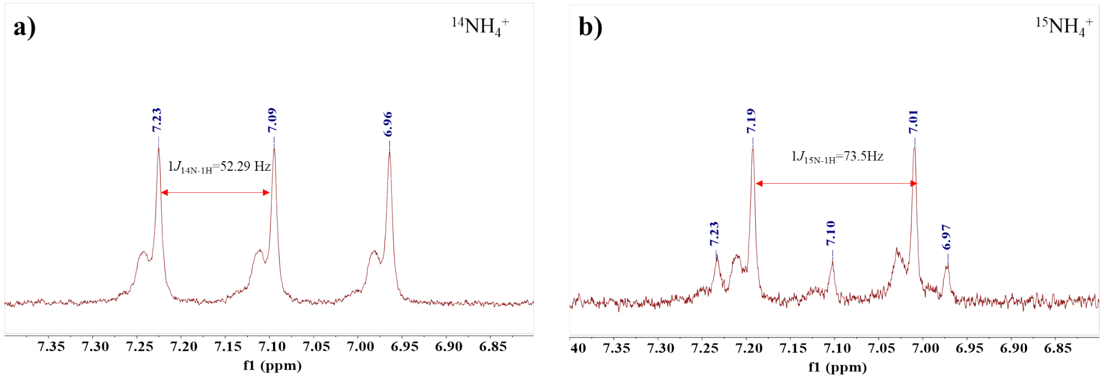

Figure S3 ^1H NMR spectrum of NH_4SO_4 evolved from **(a)** $^{14}N_2$ and **(b)** $^{15}N_2$ hydrogenation using Ru_2@ST catalyst. Reaction condition: 350°C, 0,1MPa and light irradiation (1080 w m^{-2}).

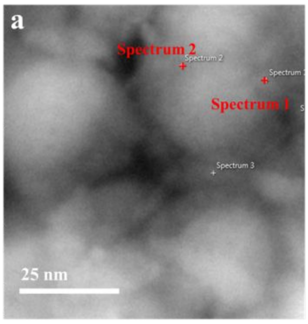

Spectrum 1	Line Type	k Factor	Wt%	Wt% Sigma
N	K series	3.515	0	0
Ti	K series	1.09	35.61	1.77
Sr	K series	2.544	48.59	2.38
Ru	K series	6.418	0	0
Cs	K series	47.34	15.8	3.95
Total:			100	

Spectrum 2	Line Type	k Factor	Wt%	Wt% Sigma
Ti	K series	1.09	28.9	1.15
Ni	K series	1.164	23.7	0.97
Sr	K series	2.544	41.75	1.62
Ru	K series	6.418	1.45	0.64
Cs	K series	47.34	4.21	3.19
Total:			100	

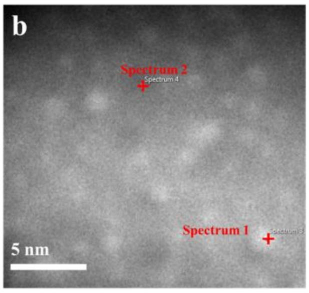

Spectrum 1	Line type	K factor	Wt%	Wt% sigma
O	K series	2.02	42.33	2.34
Ti	K series	1.09	14.12	0.97
Sr	K series	2.544	16.26	1.34
Ru	K series	6.418	12.39	2.02
Cs	K series	47.34	14.89	0.67
Total:			100	

Spectrum 2	Line type	K factor	Wt%	Wt% sigma
O	K series	2.02	38.59	1.86
Ti	K series	1.09	25.38	1.35
Sr	K series	2.544	36.03	1.94
Ru	K series	6.418	0	0
Cs	K series	47.34	0	0
Total:			100	

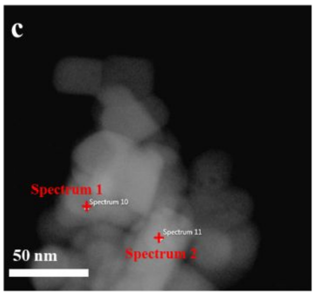

Spectrum 1	Line Type	k Factor	Wt%	Wt% Sigma
O	K series	2.02	23.08	0.8
Ti	K series	1.09	25.58	0.67
Sr	L series	1.726	49.7	1
Cs	L series	2.015	1.64	0.63
Total:			100	

Spectrum 2	Line Type	k Factor	Wt%	Wt% Sigma
O	K series	2.02	26.05	0.55
Ti	K series	1.09	24.1	0.43
Sr	L series	1.726	48.9	0.66
Cs	L series	2.015	0.96	0.4
Total:			100	

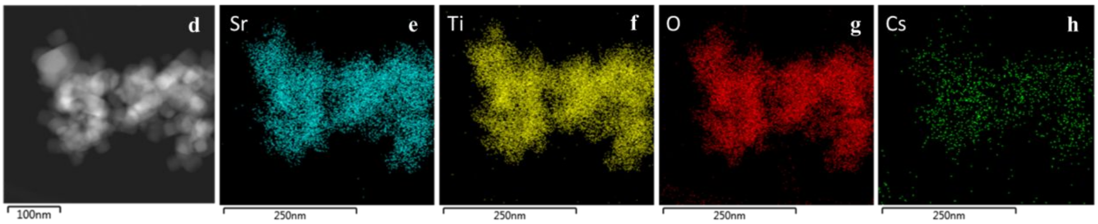

Figure S4 ADF-STEM image obtained from $Cs_{10}Ru_2$@ST sample and EDS results acquired from the high contrast point (spectrum 1) and low contract point (spectrum 2) before **(a)** and after the hydrogen activation **(b)**. **(c)** ADF-STEM image obtained from Cs@ST sample after activation, and the EDS results from two random points in the sample. (**d-h**) Elemental mapping of a representative STEM image of Cs@ST.

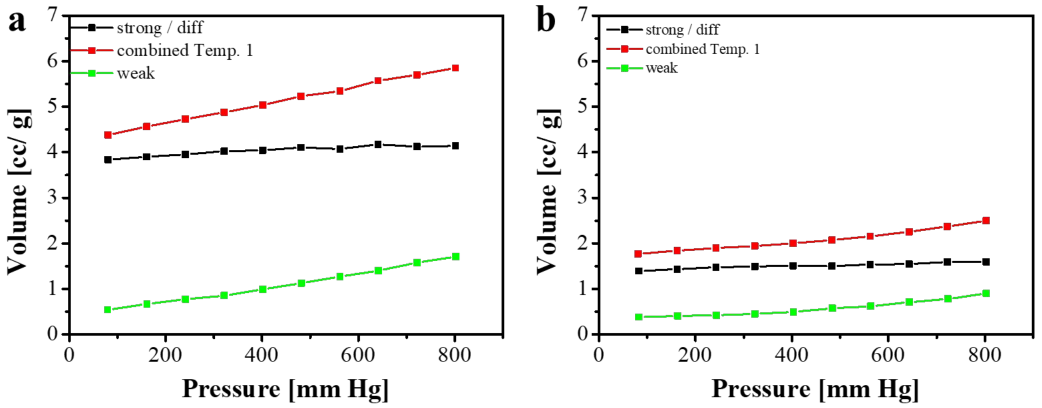

Figure S5. CO chemisorption plots obtained from (a) Ru_2@ST and (b) $Cs_{10}Ru_2$@ST.

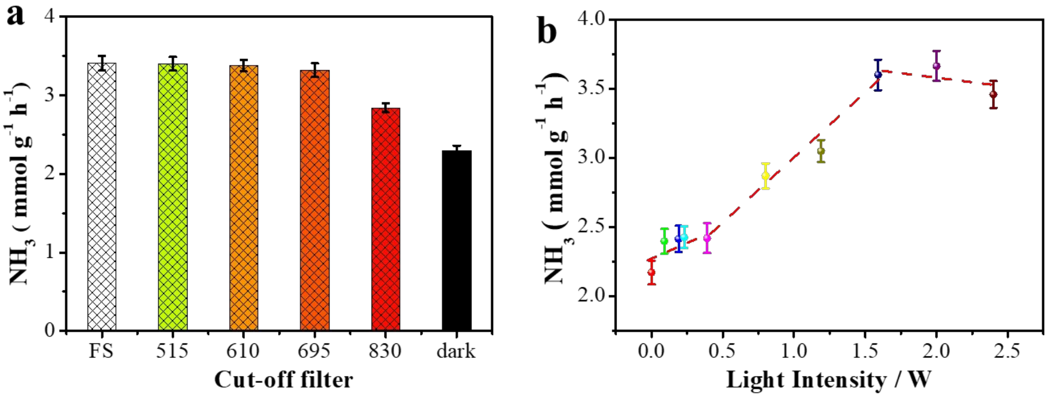

Figure S6. (a) NH_3 production rates obtained by altering different cut-off filters. light source, 300 W Xenon lamp with FS 1080 W/m^2, **(b)** NH_3 production rates observed by using monochromatic 980 nm laser as the light source with different light intensity. Reaction conditions: 50 mg $Cs_{10}Ru_2$@ST, 10 mL min^{-1} N_2 + 30 mL min^{-1} H_2, 350°C and 0.1 Mpa.

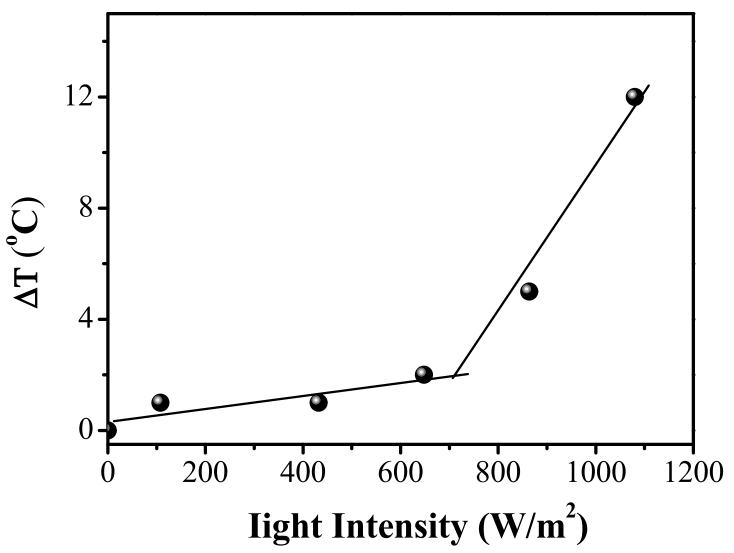

Figure S7. Temperature variation with light intensity at the catalyst surface.

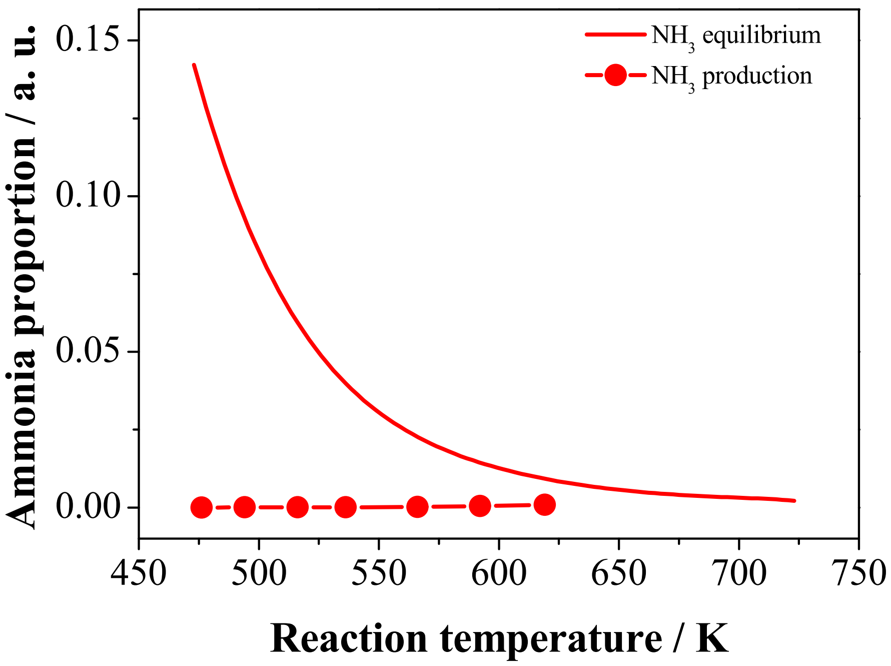

Figure S8. Ammonia proportion in the equilibrium as a function of the temperature (red line without dots)[2] and the concentration of ammonia at different reaction temperature under light irradiation (red circles) reported in this work. Conditions: Calculated based on stoichiometric N_2 and H_2 amounts at atmospheric pressure.

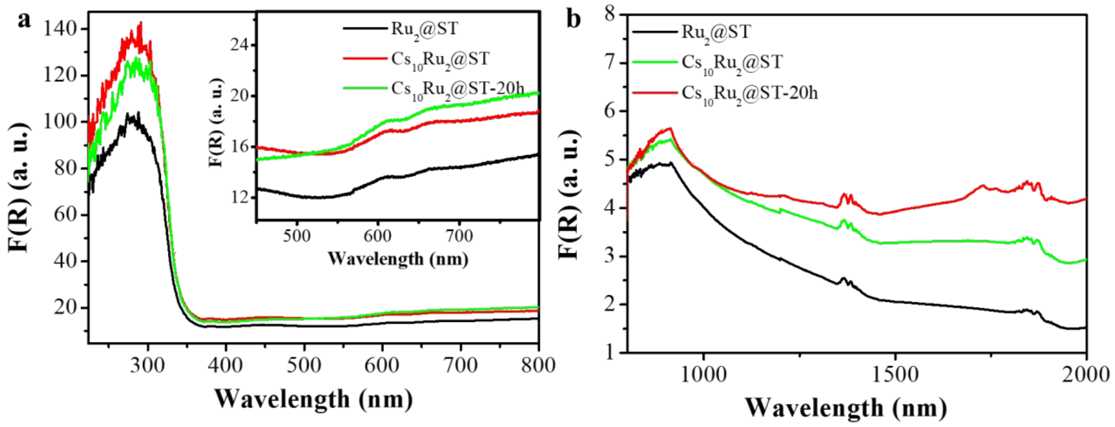

Figure S9 Diffuse reflectance UV-Vis (a) and NIR (b) spectra, plotted as the Kubelka-Munk function of the reflectance (R) of ST (blue), Ru_2@ST (black), $Cs_{10}Ru_2$@ST (red) and $Cs_{10}Ru_2$@ST after 120h reaction (green). Panel a) inset shows enlarged spectrum from range 450 to 800 nm.

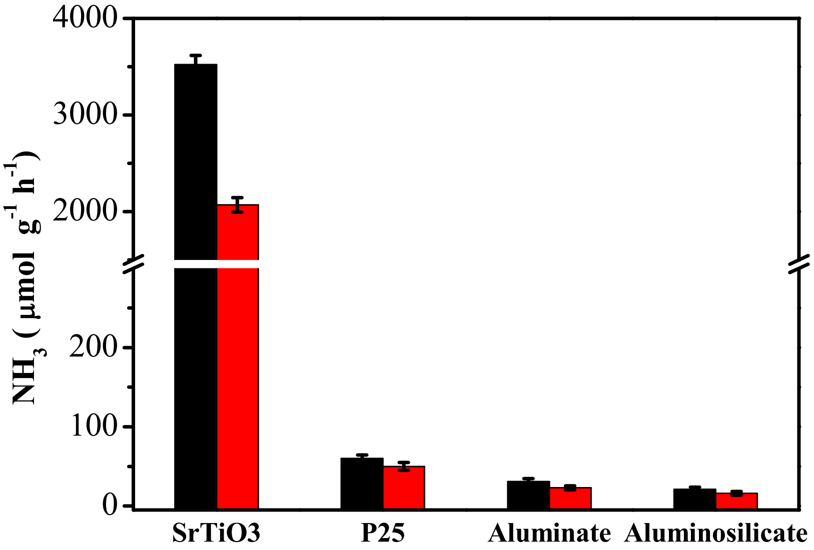

Figure S10 NH_3 production rate as substrate function. Dark bars correspond to experiments under light irradiation, while red bars correspond to dark conditions. Error bars correspond to standard deviation.

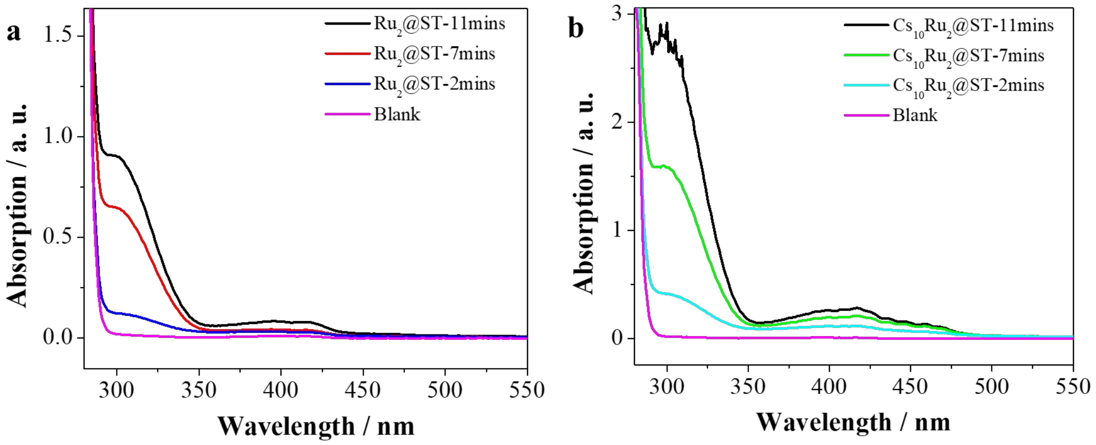

Figure S11 UV-Vis absorption spectra of TCNE solution (0.1 mM) in acetonitrile in contact with $Cs_{10}Ru_2$@ST **(a)** and Ru_2@ST (b) for different time.

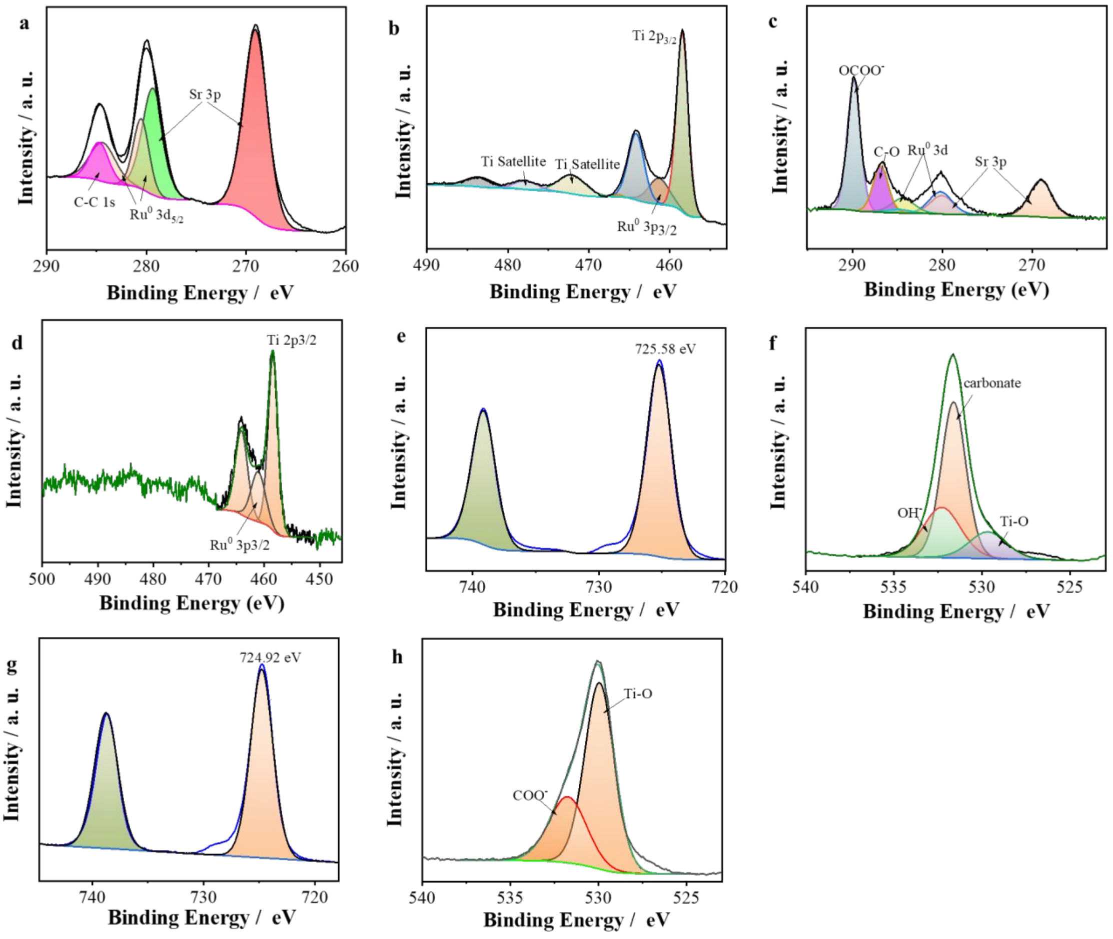

Figure S12 High resolution XPS spectrum of **(a)** Ru 3d + C1s + Sr 3p spectra and **(b)** Ti 2p + Ru 3p spectra acquired from Ru_2@ST sample after the activation; **(c)** Ru 3d + C1s + Sr 3p spectra, **(d)** Ti 2p + Ru 3p and **(e)** Cs 3d **(f)** O 1s spectra acquired from $Cs_{10}Ru_2$@ST sample after the activation; **(g)** Cs 3d and **(h)** O 1s spectra acquired from $Cs_{10}Ru_2$@ST sample before the activation.

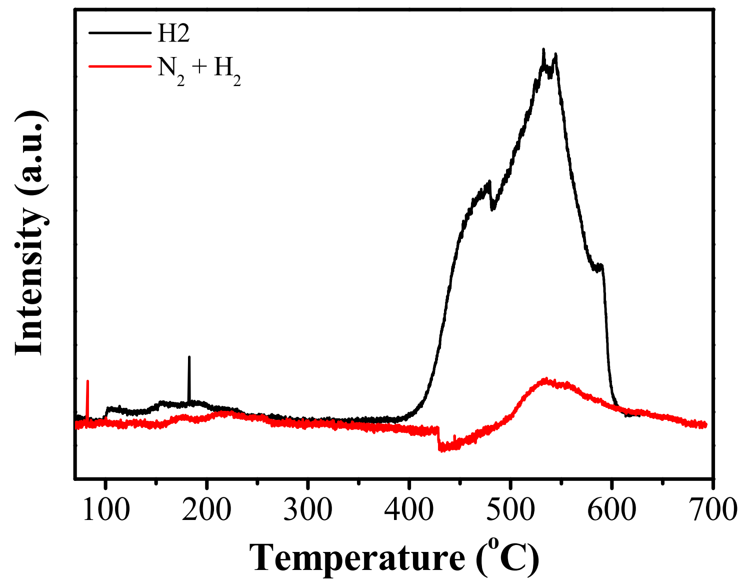

Figure S13 H_2 TPD profiles of $Cs_{10}Ru_2$@ST samples after activation in H_2 atmosphere at 350 °C (black) and after activation and sequential N_2 and H_2 flow at 350 °C (red).

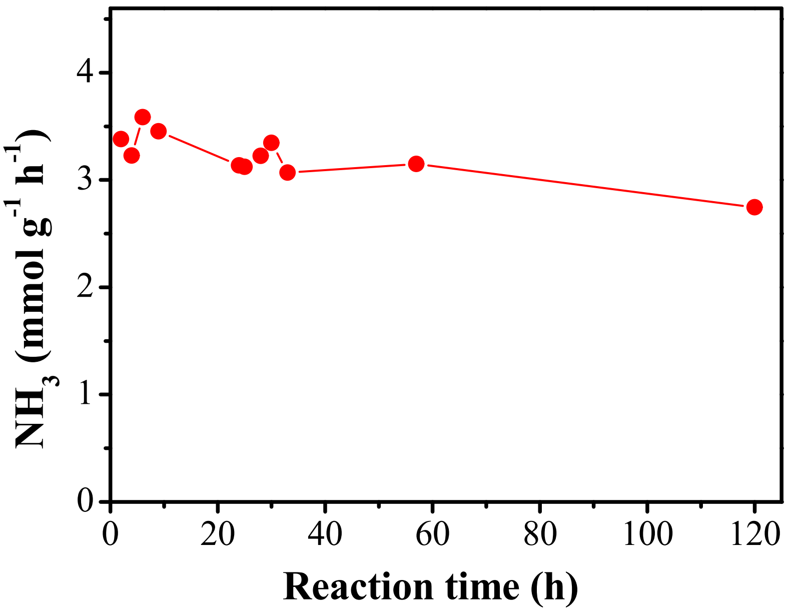

Figure S14 NH_3 production rate from $Cs_{10}Ru_2$@ST at 350 °C under light irradiation (1080 W m^{-2}) for 120 h. Reaction conditions: 60 mg catalyst; 10 mL min^{-1} N_2 + 30 mL min^{-1} H_2; 350 °C; 0.1 Mpa.

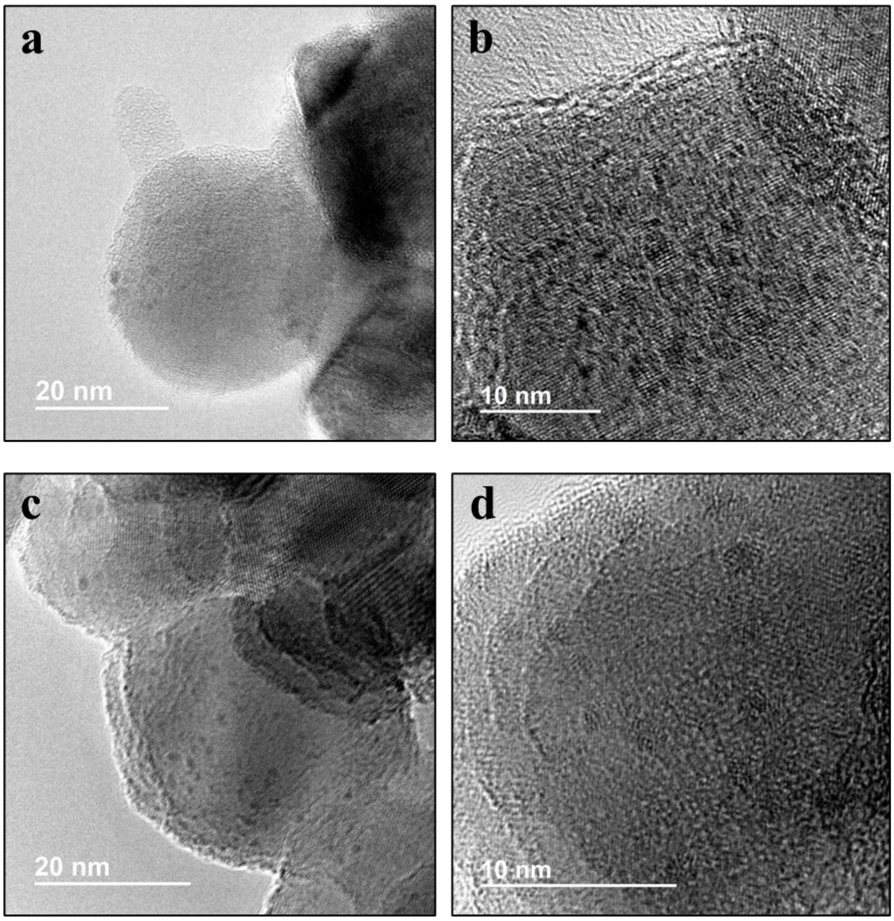

Figure S15 Representative HR-TEM images of $Cs_{10}Ru_2$@ST photocatalysts before the reaction (**a** and **b**) and after 120 h reaction at 350 °C under light irradiation (1080 W m^{-2}) (**c** and **d**).

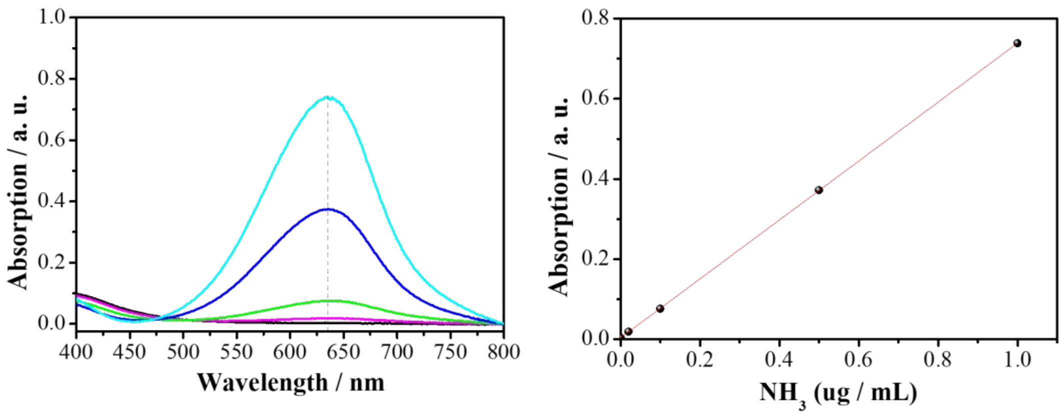

Figure S16 UV-vis absorption spectra of indophenol blue **(a)** and calibration curve **(b)** from standard NH_4Cl solution.

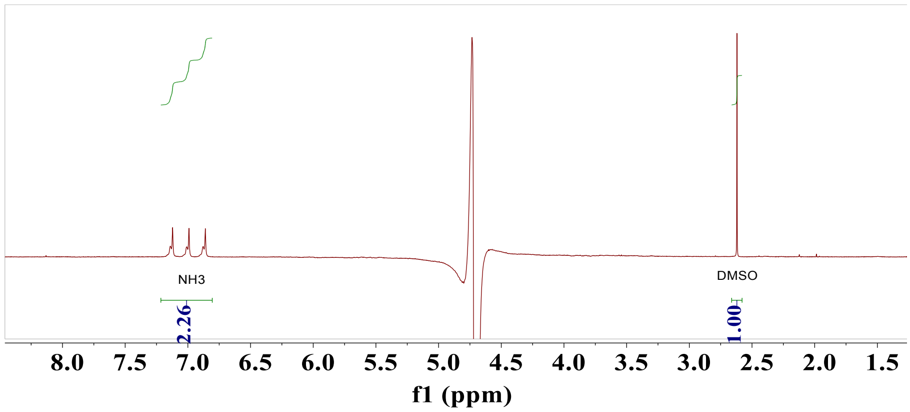

Figure S17. Representative ^{1}H NMR spectrum for the quantitative NH_3 determination with DMSO as the internal standard

Table S1. Summary of some of the most efficient catalysts for N_2 hydrogenation reported in the literature, including metal loading, conditions and reaction rate.

Entry	Catalyst	Metal Loading (wt%)	Conditions	Reaction rate ($\mu mol \cdot g^{-1} \cdot h^{-1}$)	Reference
1	**$Cs_{10}Ru_2$@ST**	Ru:2.6, Cs: 13	350°C 0.1Mpa	2600	***This work***
2	**$Cs_{10}Ru_2$@ST**	Ru:2.6, Cs: 13	350°C 0.1Mpa Xenon lamp irradiation	3580	***This work***
4	**Ni@LaN NPs**	Ni: 12.5	340°C 0.1Mpa	2665	3
5	**Cs-Co_3Mo_3N**	Cs: 36.9	400°C 0.1Mpa	986	4
6	**$Ru/C12A7{:}e^{-}$**	Ru: 1.8	340°C 0.1Mpa	2021	5
7	**Cs-Ru/MgO**	Ru: 2.0	340°C 0.1Mpa	3200	5

8	**Ru/$SrTiO_3$**	Ru: 2.0	400^oC 0.1Mpa	774	6
9	**Co/C12A7:e^{-e}**	Co: 2.6	400^oC 0.1Mpa	912	7
10	**LaRuSi**	-	400^oC 0.1Mpa	1760	8
11	**LaCoSi**	-	400^oC 0.1Mpa	1250	9
12	**Cs-Ru/MgO**	Ru 2.5wt% Cs:Ru=2:1	333^oC 0.1 Mpa LED 4.7 W/Cm^2	4464	10

References

1 Tzollas, N. M.; Zachariadis, G. A.; Anthemidis, A. N.; Stratis, J. A., A new approach to indophenol blue method for determination of ammonium in geothermal waters with high mineral content. *Int. J. Environ. Anal. Chem*. **2010**, 90, 115-126.

2 M. Martín Martín, *Industrial Chemical Process Analysis and Design*, **2016**, Pages 199-297

3 Ye, T.-N.; Park, S.-W.; Lu, Y.; Li, J.; Sasase, M.; Kitano, M.; Tada, T.; Hosono, H., Vacancy-enabled N_2 activation for ammonia synthesis on an Ni-loaded catalyst. *Nature* **2020,** 583, 391-395..

4 Kojima, R.; Aika, K.-i., Cobalt molybdenum bimetallic nitride catalysts for ammonia synthesis: Part 2. Kinetic study. *Appl. Catal. A-GEN,* **2001,** 218, 121-128.

5 M. Kitano, Y. Inoue, H. Ishikawa, K. Yamagata, T. Nakao, T. Tada, S. Matsuishi, T. Yokoyama, M. Hara, H. Hosono, Essential role of hydride ion in ruthenium-based ammonia synthesis catalysts. *Chem. Sci.* **2016**, 7, 4036.

6 Horiuchi, Y.; Kamei, G.; Saito, M.; Matsuoka, M., Development of Ruthenium-loaded Alkaline-earth Titanates as Catalysts for Ammonia Synthesis. *Chem. Lett.* **2013,** 42, 1282-1284..

7 Inoue, Y.; Kitano, M.; Tokunari, M.; Taniguchi, T.; Ooya, K.; Abe, H.; Niwa, Y.; Sasase, M.; Hara, M.; Hosono, H., Direct Activation of Cobalt Catalyst by

$12CaO{\cdot}7Al_2O_3$ Electride for Ammonia Synthesis. *ACS Catal.* **2019,** 9, 1670-1679.

8 Wu, J.; Li, J.; Gong, Y.; Kitano, M.; Inoshita, T.; Hosono, H., Intermetallic Electride Catalyst as a Platform for Ammonia Synthesis. *Angew. Chem. Int. Ed.* **2019,** 58, 825-829.

9 Gong, Y.; Wu, J.; Kitano, M.; Wang, J.; Ye, T.-N.; Li, J.; Kobayashi, Y.; Kishida, K.; Abe, H.; Niwa, Y.; Yang, H.; Tada, T.; Hosono, H., Ternary intermetallic LaCoSi as a catalyst for N_2 activation. *Nat. Catal.* **2018,** 1, 178-185.

10 Li, X.; Zhang, X.; Everitt, H. O.; Liu, J., Light-Induced Thermal Gradients in Ruthenium Catalysts Significantly Enhance Ammonia Production. *Nano Lett.* 2019, 19, 1706-1711.
